# Energy Guidance Using Indirect Calorimetry for Intestinal Failure Patients with Home Parenteral Nutrition: The Right Bag Right at the Start

**DOI:** 10.3390/nu15061464

**Published:** 2023-03-17

**Authors:** Zenzi Rosseel, Pieter-Jan Cortoos, Elisabeth De Waele

**Affiliations:** 1 Department of Pharmacy, Universitair Ziekenhuis Brussel (UZB), 1090 Brussels, Belgium; zenzi.rosseel@uzbrussel.be (Z.R.); pieterjan.cortoos@uzbrussel.be (P.-J.C.); 2 Department of Clinical Nutrition, Universitair Ziekenhuis Brussel (UZB), 1090 Brussels, Belgium; 3 Faculty of Medicine and Pharmacy, Vrije Universiteit Brussel (VUB), 1090 Brussels, Belgium; 4 Department of Intensive Care, Universitair Ziekenhuis Brussel (UZB), 1090 Brussels, Belgium

**Keywords:** intestinal failure, home parenteral nutrition, indirect calorimetry, resting energy expenditure

## Abstract

Intestinal failure is defined as the inability to absorb the minimum of macro and micronutrients, minerals and vitamins due to a reduction in gut function. In a subpopulation of patients with a dysfunctional gastrointestinal system, treatment with total or supplemental parenteral nutrition is required. The golden standard for the determination of energy expenditure is indirect calorimetry. This method enables an individualized nutritional treatment based on measurements instead of equations or body weight calculations. The possible use and advantages of this technology in a home PN setting need critical evaluation. For this narrative review, a bibliographic search is performed in PubMed and Web of Science using the following terms: ‘indirect calorimetry’, ‘home parenteral nutrition’, ‘intestinal failure’, ‘parenteral nutrition’, ‘resting energy expenditure’, ‘energy expenditure’ and ‘science implementation’. The use of IC is widely embedded in the hospital setting but more research is necessary to investigate the role of IC in a home setting and especially in IF patients. It is important that scientific output is generated in order to improve patients’ outcome and develop nutritional care paths.

## 1. Introduction

Patients with a dysfunctional gastrointestinal system, for example, intestinal failure (IF), can be malnourished when the physiological demand of nutrients is higher than the nutritional intake [1]. IF is characterized by malabsorption leading to diarrhea and dehydration, making IF one of the indications for treatment with parenteral nutrition (PN) [2]. If such patients are deemed physically and emotionally stable and have support from a multidisciplinary team, they can be discharged with home parenteral nutrition (HPN). HPN is an embedded, well-known and widespread treatment in many countries. One of the most challenging features of HPN treatment is the search for a well-tolerated PN mixture that fulfils the nutritional requirements [3], next to minimizing and preventing PN complications such as refeeding syndrome (RFS) and catheter-related bloodstream infections (CRBSI) [4]. In particular, parenteral nutrition-associated liver disease (PNALD) and the occurrence of hyperglycemia can be life-threatening and are associated with macronutrient dosing [5,6,7]. The recommendations by the European Society of Clinical Nutrition and Metabolism (ESPEN) state an individualized assessment is essential [8] in order to develop a nutritional plan because of an alteration in metabolism due to IF. The current practice often uses body weight-based calculations or equations to determine caloric goals [8]. However, the use of indirect calorimetry (IC) has a proven benefit in certain patient populations such as the critically ill [9], and in many hospitals this ‘gold standard’ has become common practice [1,10]. An adequate and accurate nutritional plan is necessary to meet requirements, reduce times of recovery in the acute and chronic phases and prevent complications. The aim of this narrative review was to explore the possible benefits of the use of IC compared to predictive equations and identify possible barriers and facilitators for implementation in clinical practice including health care economic issues.

## 2. Methods

A bibliographic search in PubMed and Web of Science was conducted for this narrative review, with publications published between 2000 and 2022 included. This search was based on a combination of MeSH and free terms: ‘indirect calorimetry’, ‘direct calorimetry’, ‘home parenteral nutrition’, ‘intestinal failure’, ‘parenteral nutrition’, ‘resting energy expenditure’, ‘energy expenditure’ and ‘science implementation’. Additional papers were identified by snowballing.

## 3. Importance and Management of (H)PN in IF

As oral nutrition intolerance or oral nutrition alone cannot cover all requirements in IF patients, e.g., due to short bowel, Crohn’s disease and bowel villous atrophy, PN is frequently indicated for many years [11,12]. Several studies published in the beginning of the 21th century demonstrated that long-term HPN is a safe therapy and the primary therapeutic option for IF [13,14,15,16]. Two studies evaluated the survival rate of IF patients after a few years of HPN. After 5 and 10 years, the survival rate for individuals with SBS requiring HPN was 73% and 56%, respectively [12]. In patients with Crohn’s disease, the 3-year survival rate was 87%, 84% for individuals with ischemic bowel and 62% for patients with pseudo-obstruction [17].

A major survey in eight European countries reviewed how HPN patients were monitored after discharge in terms of recommendations, home visits and how problems were managed. At 92% of the centers, a multidisciplinary nutrition support team was available. At each visit, almost every center assessed body weight, hydration condition and oral intake. Patients could contact the HPN team in 76% of cases in case of problems or complications such as catheter sepsis, while written recommendations to monitor practice were accessible in 66%. Just 21% of the facilities examined HPN patients’ skills every three months [18]. According to this survey, a dedicated HPN team is not currently standard practice. Another study in Scotland assessed the frequency and monitoring of blood, weight and anthropometry in 53 HPN patients over the course of 141 clinic assessments. A blood analysis was performed in 93% of the evaluations, a weight check in 86%, vitamins and trace elements were determined in 62% of the cases and anthropometry (arm circumferences) was performed in only 24% of the cases [19]. Both studies, however, did not mention anything about a nutritional assessment in HPN. Nevertheless, the guidelines clearly recommend the recording of nutritional assessment results and findings on physical examination in medical records for nutritional support [20]. Consequently, more effort should be made in documenting and performing nutritional assessments in HPN.

## 4. Nutritional Assessment Methods

### 4.1. Predictive Equations, Low Accuracy Alternative?

There are several methods to determine energy requirements and energy dosing, starting with multiple predictive equations such as those developed by Harris and Benedict and Ireton-Jones [21]. The ESPEN recommendations of 20 kcal/kg/day and the Ireton-Jones predictive equation have been found to provide the best approximation of a patient’s energy requirements in an IF population [22]. However, use of these predictive equations can result in substantial negative or positive energy balances in patients on HPN with IF, and preference is given to IC to measure the REE. To illustrate this, Edakkanambeth et al. published a case report of a 62-year-old male with a long-standing jejunocutaneous fistula. The Ireton-Jones equation varied with an underestimation of 34% from IC and the Harris–Benedict with −26%. The Owen equation (−2%) and World Health Organization (WHO) guidelines (+2%) were closest to IC-calculated REE [23]. This exemplified patient specific variance. A cross-sectional study compared the accuracy of IC with the use of predictive equations in adult patients on PN. The ‘short equation’ (54.1%) by McClave et al. and the Owen equation (46.6%) had the highest accuracy compared with Mifflin (30.7%) and Ireton-Jones (37.7%) [24].

In populations with an extreme body mass index (BMI) (BMI < 16kg/m^2^ and BMI > 40 kg/m^2^), IC was equally the first choice compared to predictive equations [25]. Several studies observed lower BMI values (16.6–21.3) in HPN patients [26,27,28,29]. Evaluating the accuracy of 18 predictive equations and IC for calculating REE in patients with underweight (BMI < 18.5), normal weight (18.5 < BMI < 30) and overweight (BMI > 30), all equations reached only an accuracy between 9–45% (BMI < 18.5), 12–52% (18.5 < BMI < 25), 39–58% (25 < BMI < 30) and 10–53% for BMI > 30 compared to IC [30]. The determination of REE should therefore be made by IC [24] because no equation was accurate enough in comparison [30,31].

### 4.2. Direct Calorimetry

Direct calorimetry (DC) was developed prior to indirect calorimetry. Where the indirect calorimeter measures oxygen consumption and carbon dioxide production, the direct calorimeter technique is based on measuring generated heat [32,33].

Between 1900 and 1940, tests to validate DC were conducted on medium-to-large mammals. In the original DC setup, guinea pigs were kept in ice-coated cages with the heat produced by the guinea pig causing the ice to melt. The amount of ice that melted was proportional to the amount of heat released, in accordance with the law of latent heat. These tests were to be carried out during wintertime for ambient temperatures not to affect the rate of melting. At the beginning of the 20th century, direct (by direct measurement of heat production) and indirect (measurement by gas analysis) calorimetry took form and showed almost perfect correlation [32]. DC remains the gold standard for measuring human metabolic rate but is impractical in a clinical setting [34]. From the 1920s on, IC could be used in clinical medicine, and continued to be optimized in the following century [35].

### 4.3. IC Technology

Indirect calorimeters are designed to determine REE in a clinical setting such as patients on mechanical ventilation as well as in patients who breathe spontaneously. It is a non-invasive, easy-to-use technique measuring the oxygen consumption and the carbon dioxide production [35]. These parameters are then introduced in the Weir equation (EE (kcal/day) = 1440 × [3.94 × VO_2_ (mL/min) + 1.11 × VCO_2_ (mL/min) + urinary nitrogen (g/day) × 2.17]) [10] which allows for the calculation of REE [35]. An IC can be carried out in spontaneously breathing patients as such is the case in HPN by the use of a canopy, or can be connected to the ventilator in mechanically ventilated patients [35]. A 30-min measurement is usually appropriate to obtain reliable results, but if a steady condition is established, 10–15 min can already be sufficient [36]. Patients with IF on HPN can undergo this testing without major issues.

By the use of IC, the health care professional will be able to provide a nutritional care plan based on the measured energy requirements and calculated protein needed to reduce protein breakdown due to catabolism, enhance recovery and facilitate muscle preservation [10].

### 4.4. IC Added Value

IC can be performed in spontaneously breathing, stable patients and can be used in weight modulation approaches [37,38]. By determining the REE, weight loss or gain can be expected based on a negative or positive energy balance. The determination of REE in combination with body composition and body weight can be used for further optimization of the nutritional prescription [36]. In recent years, the use of IC has been extensively encouraged in different patient populations because various studies have shown that both under- and overfeeding can have detrimental effects.

A retrospective analysis of almost 1200 ICU patients was performed to evaluate the administered calories relative to the measured REE and a possible association with 60-day mortality. Achieving 70% of the administrated calories/REE was associated with a lower mortality, while percentages exceeding 100% were associated with higher mortality [39]. The usage of IC provides information about energy requirements, but nothing can be concluded about the protein requirements. Because the energy/protein ratio is present in a fixed ratio in ready-to-use mixtures, it is important to select the right bag right at the start of PN treatment.

Numerous studies comparing IC versus standard nutritional care have evaluated different endpoints. Many of these studies involved ICU patients, who are completely different from IF patients, yet the added value of using IC in this patient population is no longer debatable.

The TICACOS trial included 417 patients from seven different centers and sought to determine the added value of measuring REE on a daily basis. Tight calorie control guided by the use of IC did not significantly reduce the infection rate and mortality [40].

The EAT-ICU trial included 199 ICU patients and compared early goal-directed nutrition with the standard of care. Patients in the early goal-directed nutrition group received more energy and proteins since their intake was based on IC-guided assessments. On the other hand, more episodes of hyperglycemia were observed [41]. A systemic review with eight randomized controlled trials (RCT) involving 991 adult ICU patients concluded that short-term mortality was significantly lower in the IC group, while it did not affect the duration of mechanical ventilation, LOS in ICU and in hospital in general. These findings encourage the use of IC-guided energy delivery in ICU patients [9] and reinforce the ESPEN grade A recommendations. ESPEN provides guidelines regarding home parenteral nutrition, but these are not specific for IF patients.

### 4.5. Disadvantages of IC

In ICU patients, the use of IC can be considered the ‘gold standard’ [9,36], but there are still several disadvantages that might affect the use, outcomes and consequently nutritional management. A study about IC in clinical practice addressed a few issues such as air leakages in the respiratory circuit and a patient population where the use of IC was not possible: spontaneously breathing patients with oxygen support [35,42]. As an IC can be performed with a canopy in spontaneously breathing patients or connected to the ventilator [35], it is important to minimize air leaks whenever possible [43]. It is essential, before using the IC, to respect the warm-up time, calibration phase and steady-state period. The warm-up time and calibration phase are determined by the manufacturer. A steady-state period is defined as a variation in VCO_2_ and VO_2_ of <10% for at least five consecutive minutes. A steady state could be obtained in a three- or four-minute interval in ambulatory patients or healthy volunteers [43,44]. The respiratory coefficient (RQ) is the ratio of carbon dioxide production to oxygen consumption (VCO_2_/VO_2_). A reliable result is obtained when the RQ is between 0.67 and 1 [36,45]. Disinfection of non-disposable material after every measurement is necessary for infection prevention.

Health care professionals acquire education and undergo routine retraining under the direction of approved procedures in order to perform reliable measurements. With the use of such technology, both acquisition costs and recurrent costs such as disposables are inevitable. Health care cost analyses revealed that in the critically ill patients, the use of IC was cost-beneficial by reducing the risk of infections associated with under- and overfeeding [46].

## 5. IC from Bench to Bedside

### 5.1. Use of IC in HPN Patients

Much research was generated over the past few decades concerning the use and applications of IC. Between 1918 and 2022, 8.734 PubMed references were found with 5.657 hits between 2002 and 2022. Sixty-five percent of all research concerning IC was therefore conducted in the last two decades. It is encouraging to see that the global use of IC is being explored in different patient populations. A lot of data are generated from IC in critically ill patients, but data are scarce in other patient populations [10,31,35,47]. Not many studies evaluated the use of IC in the home setting, so this needs to be investigated on a larger scale.

### 5.2. Protocols

**Figure 1:**
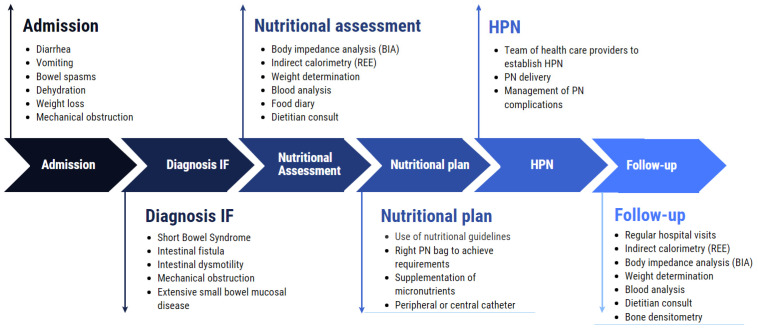
Different steps, from hospital admission to discharge and follow-up with HPN. Protocol used in UZ Brussel.

Based on the research and literature, it is important to develop protocols and care paths including the use of IC, detailing how HPN patients need to be initiated and followed-up after discharge. A practical implementation of the use of IC will enable the registration and generation of data about IC use in IF patients on HPN. Figure 1 shows the protocol used in IF patients treated in our hospital in Brussels, Belgium. It is based on ESPEN recommendations and describes the care path from admission with IF symptoms until hospital discharge with HPN [7].

According to surveys and guidelines, HPN patients in general are to be surrounded by well-trained health care providers and to visit the hospital on a regular basis for the evaluation of nutritional status and possible interventions to enhance patients’ outcomes [8,18,48]. Since IF patients’ metabolisms change over time and vary in between patients [1], it is important to define REE at start-up and redefine during follow-up, particularly in long-term (>6 months) HPN patients. This allows for an individualized energy dosing plan. Patients can consult doctors or dietitians who are qualified to perform IC.

### 5.3. Implementation Science

Patients derive no benefit from all this scientific output if it is not translated into clinical recommendations and finally clinical practice. This translation requires time and effort and depends on the health care providers’ attitudes, expertise, behavior and various external factors such as local organization and available resources [49,50,51]. ‘Implementation science’ addresses the challenges of translating research output into clinical practice, from bench to bedside [50].

Over the last 21 years, the principles of translating scientific output into clinical practice have gained tremendous momentum. Between 1945 and 2022, the MeSH term ‘Implementation Science’ in PubMed yielded 123.190 hits, with 118.156 hits between 2002 and 2022. A lot of research has already been done in the field of science implementation regarding nutrition (with 6.031 hits on PubMed, of which 95% have been published in the last 20 years), but less has been found regarding PN and on the use of IC in HPN, where no publications are available. Generating additional scientific output would allow for the development of guidelines and therefore better position the use of IC [52]. The main goal of such scientific work and output is to offer better treatments, reduce morbidity and mortality and thereby reduce economic health burden [50]. Two studies, both of which were conducted in ICU patients receiving EN, showed how science implementation might enhance patients’ outcomes. The first quality improvement project involved 49 ICU patients and attempted to show that higher protein EN formulations could deliver >80% of the protein requirements.

They found that enriched protein EN formulations were well-tolerated in a limited ICU group since three out of four patients reached that target [53]. The second enhancement project evaluated the impact of the presence of a registered dietician nutritionist (RDN) on EN compliance and delivery in 50 ICU patients. These privileges improved EN compliance and protein delivery [54].

### 5.4. IC Implementation on a Larger Scale

In our own center, we have many years of expertise on the use of IC in various patient populations, which might help to implement the correct use of IC on a larger scale.

Next to the already-mentioned advantages or strengths of IC, we perceive the following opportunities, weaknesses and threats.

Opportunities arise for conducting pre- and post-studies to see whether there is a difference in outcome or other parameters such as change in energy delivery, liver test disturbances or infection rate between HPN patients in whom an IC was performed and in HPN patients in whom guidelines were consulted or nutritional needs were calculated using predictive equations. Based on these findings, it is possible to develop protocols that clearly outline how an HPN patient is followed-up before and after discharge with HPN. The exchange of this data and experience will increase the use of IC worldwide, in case of a positive evaluation.

Contact with experts in the field revealed that the cost of an indirect calorimeter is a major barrier to its implementation, and hence data generating [35]. Equally, there is a need for compact, portable and easy-to-use indirect calorimeters for use in an ambulatory setting. The Breezing^®^ device, a pocket-sized IC potentially suitable for remote patient monitoring [55,56], is currently still under development but has showed reliable results in healthy subjects. Hands-on training will be necessary for its correct implementation in a clinical and home setting. Correct implementation and interpretation can result in improved nutritional management and potentially in better patient outcomes. Increasing performance comes at a cost. Not only is the price a potential issue, but so is allowing time for training and performing measurements. It is important that health care providers are properly trained in order for indirect calorimeters to be used effectively and results to be accurately interpreted.

We can conclude that there is a need for large prospective trials evaluating IC use in HPN patients in order to develop protocols and provide high-level training that ensure the correct use of an indirect calorimeter.

## 6. Dietary Recommendations

Patients suffering from IF experience significant losses of macro- and micronutrients as well as inadequate gastrointestinal absorption. It is therefore important to adapt the diet to the characteristics of the remaining bowel, which macronutrients are still tolerated and where artificial substitution will be necessary [57,58]. To be able to substitute normal food, both quantitative and qualitative aspects need to be respected. Total protein and energy delivery make a part of this quantitative challenge, whereas qualitative aspects should comprise the type of lipids, type of amino acids, protein profiling and carbohydrates [8]. Protein profiling may be used to determine optimal amino acids ratios, while energy needs are based on the resting energy expenditure (REE) [59]. These energy requirements are disease-specific and need to be evaluated in each patient individually. For stable IF patients on HPN, recent guidelines recommend an intake of 20–35 kcal/kg/day [8]. REE also depends on the patients’ condition and body composition. Post-surgery patients and patients in stress have higher measured REE values [10].

### Macro- and Micronutrients

An adequate energy provision is necessary to counter macro- and micronutrients’ deficits, facilitate post-op recovery and avoid weight losses [8,60,61,62]. In an ideal situation, energy requirements are determined by the use of IC, and if that is not feasible, by the use of the European Society of Parenteral and Enteral Nutrition (ESPEN) guidelines. The ESPEN, the Australian Society of Parenteral and Enteral Nutrition (AuSPEN) and the British Association of Parenteral and Enteral Nutrition (BAPEN) all recommend an intake of 20–35 kcal/kg/day in patients with IF. The American Society for Parenteral and Enteral Nutrition (ASPEN) recommendations, on the other hand, recommend 25 kcal/kg/day or 2000 kcal daily [8,63,64].

The AuSPEN guidelines recommend lipid intake as one-third of the total energy provision [63], while the ESPEN guidelines recommend 0.8–1.5 g/kg/day lipid intake.

In terms of protein provision, the ESPEN guidelines recommend an average protein intake of 0.8–1 g/kg/day [11] in healthy individuals, compared to HPN, where the recommended intake is up to 0.8–1.4 g/kg/day, in line with the AuSPEN guidelines [63].

Protein requirements depend on the physiological state of the patient. A higher protein intake is recommended in patients with protein-losing enteropathy, high stoma losses or post-surgery for better wound healing. Little is known regarding protein dosing in IF patients, hence the ESPEN recommends individual determination [8]. Carbohydrates account for a high amount of the overall energy required by a patient and are often administrated in the form of glucose. Carbohydrates are also responsible for glycemia levels and in high amounts for hyperglycemia with detrimental effects [6,65,66].

Not only macronutrients but also micronutrients such as vitamins and trace elements (TE) are poorly absorbed. Micronutrients are essential components and act as cofactors in the human metabolism. It is therefore important to track changes in vitamins and TE requirements in patients on long-term (>6 months) HPN with IF. Standards for specialized nutrition support describe a daily addition of vitamins and TE to the PN regarding renal and hepatic impairment [20]. Although the ESPEN HPN guidelines recommend a routine evaluation of serum blood concentrations for micronutrients every 6 months [67], a 2007 review recommends measurement on a yearly base [68].

IC is the first choice to measure REE. When measuring REE with IC is not feasible, the ESPEN recommendations of 20 kcal/kg/day and the Ireton-Jones predictive equation have been found to provide the best approximation of a patient’s energy requirements in an IF population [22].

## 7. Conclusions

HPN is a commonly used treatment in IF patients with a need for artificial nutrition. It is important to adapt the diet to the needs of the patient and to the length and type of intestines remaining. This may involve the use of IC because it ensures that patients are fed more appropriately, lowering the risks of over- and undernutrition, which have detrimental effects. The use of IC is widely embedded in the ICU setting, but additional research is necessary to investigate the role of IC in a home PN setting and especially in IF patients. It is important that scientific output is generated in order to improve patients’ outcomes and develop nutritional care paths.

## Data Availability

Not applicable.
